# Effectiveness of early walking training in patients after NSTEMI treated with angioplasty in the first stage of cardiac rehabilitation

**DOI:** 10.3389/fcvm.2026.1688997

**Published:** 2026-01-29

**Authors:** L. Cepicka, T. Gabrys, M. Orczyk, Z. Nowak, A. Nowak-Lis

**Affiliations:** 1 Department of Pedagogy, West Bohemia University Plzen, Pilsen, Czechia; 2 Department of Physical Education, West Bohemia University Plzen, Pilsen, Czechia; 3 American Heart of Poland Katowice, Katowice, Poland; 4 Department of Physiotherapy Katowice, J. Kukuczka Academy of Physical Education, Katowice, Poland

**Keywords:** angioplasty, cardiac rehabilitation, exercise tolerance, myocardial infarction, walking

## Abstract

The basic exercise after the patient's mobilization is walking, the effects of which are associated with numerous benefits both in terms of the patients’ physical and mental condition. The aim of the study was to evaluate the effectiveness of earlier initiation of walking training in patients after coronary angioplasty in the first stage of cardiac rehabilitation.

**Material and methods:**

50 patients after NSTEMI were examined. Based on the recruitment and after meeting the inclusion and exclusion criteria, they were randomly assigned to two groups. The first group - clinical (*n* = 25) was subjected to 5-day rehabilitation consisting of 6 training units performed twice a day in a 30-meter corridor. The second group - control (*n* = 25), performed a standard cardiac rehabilitation program. Before starting and after completing training and rehabilitation, all patients underwent echocardiography to assess left ventricular functions (LVEF%, LVEDD, LVESD) and. 6-minute walk test to determine level of physical fitness.

**Results:**

After completing the 5-day walking training, a significant increase in exercise tolerance was observed in both the clinical and control groups. In the experimental group, a significant increase in exercise tolerance was observed (distance: +184.6 m, *p* < 0.001, d = 0.82, *η*^2^ = 0.316; walking speed: +1.84 m/s, *p* = 0.032, d = 0.74, *η*^2^ = 0.501; METs: +3.07, *p* = 0.001, d = 0.69, *η*^2^ = 0.342; HR peak: +20.68 bpm, *p* < 0.000, d = 0.816, *η*^2^ = 0.662). In the control group, the improvement was small (distance: +23.2 m, *p* = 0.044, d = 0.20, *η*^2^ = 0.112; HR peak: +6.36 bpm, *p* = 0.011, d = 0.228, *η*^2^ = 0.116).

**Conclusion:**

Early walking training significantly affects the level of exercise tolerance, similarly to a standard rehabilitation program.

## Introduction

An important element of the therapy of patients after percutaneous coronary angioplasty as a result of acute coronary syndrome (ACS) is physical rehabilitation (1). It usually begins immediately after the procedure in the intensive cardiac surveillance room in accordance with the ESC, AHA and ACC guidelines (2). If, after 12–48 h of immobilization, there are no contraindications to its initiation, the patient's mobility is gradually improved. In the first stage, breathing, relaxation and dynamic exercises of small muscle groups are performed. Then, dynamic exercises of the large muscle groups are added: sitting, standing, walking. In the 4th–6th phase, attempts to walk up the stairs are encouraged. The patient's rehabilitation is carried out by monitoring the ECG recording and measurements of heart rate and blood pressure before starting exercise, at the peak of exercise and after its completion. Walking is a natural and attractive form of activity for the patient because it does not involve strenuous exercises or excessive effort. The use of walking in physiotherapy programs has a beneficial effect on both the physical and mental condition of patients. Systematically and correctly used walking training in patients undergoing cardiac rehabilitation also has a beneficial effect on physical performance and the reduction of most risk factors for cardiovascular diseases (3–5). During early hospital rehabilitation (stage I), walking training is usually introduced at the end of the program, around the fourth day. Until then, patients take a leisurely walk down the hospital corridor. But in a situation where the effectiveness of angioplasty procedures increases and, consequently, the duration of patients' stay in the hospital ward is shortened, the possibility of introducing modifications to the rehabilitation program should be considered, to bring even better results. If there are no contraindications, walking training could start the day after the procedure. In relation to standard rehabilitation procedures, such a modification would certainly result in faster achievement of full fitness and an improvement in the level of exercise tolerance.

**Table 1:** Characteristics of study groups.

Variable	Experimental group (*n* = 25)			Control group (*n* = 25)		
	Min.	Max.	X ± SD	Min.	Max.	X ± SD
Age [years]	37.0	68.0	53.48 ± 10.58	30.0	68.0	58.52 ± 7.80
Body mass [kg]	57.0	105.0	84.20 ± 13.56	45.0	118.0	79.36 ± 17.24
Body height [m]	1.58	1.82	1.73 ± 6.73	1.54	1.80	1.67 ± 7.75
BMI [kg/m^2^]	20.3	36.7	28.04 ± 4.14	18.4	43.8	28.05 ± 5.77

X, average; Min., minimum; Max., maximum; SD, standard deviation.

**Table 2:** Types of comorbidities.

Condition type	Experimental group (*n* = 25)	Control group (*n* = 25)
	*N* (%)	*N* (%)
Ischemic heart disease	19 (76%)	17 (68%)
Type 2 diabetes	2 (8%)	1 (4%)
Hyperlipidemia	19 (76%)	21 (84%)
Hypertension	8 (32%)	6 (24%)
Myocardial infarction	25 (100%)	25 (100%)

**Table 3:** Number of stents implanted.

Number	Experimental group (*n* = 25)	Control group (*n* = 25)
	*N* (%)	*N* (%)
1	17 (68%)	15 (60%)
2	7 (28%)	6 (24%)
3	1 (4%)	3 (12%)
Total	25 (100%)	25 (100%)

**Table 4:** Standard cardiac rehabilitation program after surgery.

Period I	Period II	Period III
A 1 (day 1)	A 1 (days 2–3)	A 1 (days 4–6)
Breathing exercises, isometric exercises of selected muscle groups, exercises of small muscle groups, relaxation exercises, vertical positioning, walking around the room	Exercises from period I, dynamic exercises of the upper and lower limbs, coordination exercises, a quiet walk in the corridor	Exercises from period II Walking training in the corridor (up to 200 m), climbing stairs (day 6)

**Table 5:** Modified cardiac rehabilitation program.

Period I	Period II	Period III
A 1 (day 1)	A 1 (days 2–3)	A 1 (days 4–6)
Breathing exercises, isometric exercises of selected muscle groups, dynamic exercises of small muscle groups, relaxation exercises, vertical positioning, walking around the room	Exercises from period I, dynamic exercises of the upper and lower limbs, coordination exercises, walking training in the corridor	Exercises from period II Walking training in the corridor (up to 500 m), climbing stairs (day 6)

**Table 6:** Exercise tolerance in both study groups based on the 6MWT.

Variable		Experimental group (*n* = 25)	*p*	Control group (*n* = 25)	*p*
		X ± SD		X ± SD	
Distance [m]	I 6MWT	434.00 ± 88.88	<0.001	438.24 ± 102.12	0.044
	II 6 MWT	618.56 ± 79.00	d = 0,818	461.48 ± 102.46	d = 0,198
	*Δ*	184.56	*η*^2^ = 0,316	23.24	η^2^ = 0,112
			*p* < 0.001		
Walking speed [m/s]	I 6MWT	4.34 ± 0.88	0.032	4.38 ± 1.02	0.072
	II 6 MWT	6.18 ± 0.79	d = 0,741	4.61 ± 1.02	
	*Δ*	1.84	η^2^ = 0,501	0.23	
			*p* < 0.001		
METs [ml/kg/min]	I 6MWT	8.23 ± 1.48	<0.001	8.30 ± 1.70	0.056
	II 6 MWT	11.31 ± 1.31	d = 0,689	8.69 ± 1.70	
	*Δ*	3.07	η^2^ = 0,342	0.38	
			*p* = 0.001		
Borg scale	I 6MWT	10.88 ± 2.31	<0.001	11.28 ± 1.76	<0.001
	II 6 MWT	12.84 ± 1.79	d = 0,271	12.64 ± 1.07	d = 0,244
	*Δ*	1.96	η^2^ = 0,108	1.36	η^2^ = 0,128
			*p* = 0.061		

X ± SD, arithmetic mean ± standard deviation; delta, difference between measurements before and after the test; *p*, *p*-value; I 6MWT, first six-minute walking test; II 6MWT, second six-minute walking test; d, effect size measure (Cohen's d); *η*^2^, measure of effect size.

**Table 7:** Parameters of the six-minute walk test.

Variable		Experimental group (*n* = 25)	*p*	Control group (*n* = 25)	*p*
		X ± SD		X ± SD	
SBPrest [mmHg]	I 6MWT	124.72 ± 17.62	0.306	117.44 ± 11.97	0.063
	II 6 MWT	122.32 ± 14.64		120.84 ± 9.99	
	*Δ*	2.40		3.40	
			*p* = 0.073		
DBPrest [mmHg]	I 6MWT	74.76 ± 12.10	0.307	70.60 ± 8.59	0.103
	II 6 MWT	76.52 ± 8.83		73.20 ± 7.39	
	*Δ*	1.76		2.60	
			*p* = 0.143		
SBPpeak [mmHg]	I 6MWT	131.24 ± 20.79	0.072	132.24 ± 17.71	0.058
	II 6 MWT	138.24 ± 31.96		139.00 ± 10.80	
	*Δ*	7.00		6.76	
			*p* = 0.745		
DBPpeak [mmHg]	I 6MWT	75.88 ± 12.93	0.066	74.48 ± 8.82	0.133
	II 6 MWT	79.96 ± 9.67		77.12 ± 8.55	
	*Δ*	4.08		2.64	
			*p* = 0.335		
HRrest [beats/min]	I 6MWT	70.56 ± 9.33	0.567	69.44 ± 10.90	0.221
	II 6 MWT	71.76 ± 979		72.86 ± 8.12	
	*Δ*	1.20		3.24	
			*p* = 0.296		
HRpeak [beats//min]	I 6MWT	86.44 ± 14.11	<0.001	92.68 ± 13.33	0.011
	II 6 MWT	107.12 ± 17.33		99.04 ± 11.53	d = 0,500
	*Δ*	20.68		6.36	η^2^ = 0,188
			*p* < 0.001		

SBPrest, resting systolic blood pressure; DBPrest, resting diastolic blood pressure; SBPpeak, peak systolic blood pressure; DBPpeak, peak diastolic blood pressure; X ± SD, arithmetic mean ± standard deviation; delta, difference between measurements before and after the test; *p*, *p*-value; I 6MWT, first six-minute walking test; II 6MWT, second six-minute walking test; d, effect size measure (Cohen's d); *η*^2^, measure of effect size.

**Table 8:** Echocardiographic test parameters.

Variable	Experimental group (*n* = 25)	Control group (*n* = 25)	*p*
	X ± SD	X ± SD	
LVEF[%]	54.76 ± 5.72	53.56 ± 3.70	0.417
LVESD[mm]	31.48 ± 4.74	33.56 ± 4.77	0.129
LVEDD[mm]	49.32 ± 5.71	49.92 ± 4.91	0.914

LVEF%, left ventricular ejection fraction; LVESD, left ventricular end-systolic volume; LVEDV, left ventricular end-diastolic volume; X ± SD, arithmetic mean ± standard deviation; *p*, *p*-value.

**Table 9:** Correlation between ejection fraction and 6MWT.

Variable		Experimental group (*n* = 25)	Control group (*n* = 25)
LVEF%	*Δ*Distance [m]	*p* = 0.959	*p* = 0.665
		*r* = −0.010	*r* = 0.091
	*Δ*Walking speed [m/s]	*p* = 0.986	*p* = 0.683
		*r* = −0.003	*r* = 0.085
	*Δ*METs [ml/kg/min]	*p* = 0.986	*p* = 0.701
		*r* = −0.003	*r* = 0.080
	*Δ*HR_peak_ [beats//min]	*p* = 0.597	*p* = 0.245
		*r* = −0.110	*r* = 0.240

All data are presented as a difference (*Δ* – delta), *p* – *p*-value (*p* ≤ 0.05 was considered statistically significant), *r* – correlation coefficient.

**Table 10:** Correlation between left ventricular end-systolic dimension and 6MWT.

Variable		Experimental group (*n* = 25)	Control group (*n* = 25)
LVESD [mm]	*Δ*Distance[m]	*p* = 0.227	*p* = 0.222
		*r* = −0.250	*r* = 0.253
	*Δ*Walking speed [m/s]	*p* = 0.223	*p* = 0.223
		*r* = −0.252	*r* = 0.252
	*Δ*METs [ml/kg/min]	*p* = 0.223	*p* = 0.203
		*r* = −0.2523	*r* = 0.263
	*Δ*HR_peak_ [beats//min]	*p* = 0.705	*p* = 0.649
		*r* = 0.079	*r* = −0.095

All data are presented as a difference (*Δ* – delta), *p* – *p*-value (*p* ≤ 0.05 was considered statistically significant), *r* – correlation coefficient.

**Table 11:** Correlation between left ventricular end-diastolic dimension and 6MWT.

Variable		Experimental group (*n* = 25)	Control group (*n* = 25)
LVEDD [mm]	*Δ*Distance[m]	*p* = 0.954	*p* = 0.548
		*r* = 0.011	*r* = 0.126
	*Δ*Walking speed [m/s]	*p* = 0.932	*p* = 0.520
		*r* = 0.017	*r* = 0.134
	*Δ*METs [ml/kg/min]	*p* = 0.932	*p* = 0.519
		*r* = 0.017	*r* = 0.135
	*Δ*HR_peak_ [beats//min]	*p* = 0.539	*p* = 0.552
		*r* = −0.414	*r* = 0.124

All data are presented as a difference (*Δ* – delta), *p* – *p*-value (*p* ≤ 0.05 was considered statistically significant), *r* – correlation coefficient.

Despite the growing number of publications on early cardiac rehabilitation, there remains a clear research gap regarding the very early implementation of walking training, initiated as early as the second day after percutaneous coronary intervention (PCI) in low-risk NSTEMI patients. Available studies focus primarily on standard protocols in which more intensive mobilization is introduced only from the fourth day, which does not reflect contemporary clinical practice associated with progressively shorter hospital stays.

Early mobilization of patients after acute coronary syndromes (ACS) and percutaneous coronary interventions (PCI) is currently considered safe and beneficial, provided it is conducted in a clinical setting and in accordance with established standards.

However, there are situations that pose increased risk and require careful assessment before initiating physical training during Phase I rehabilitation. These include: hemodynamic instability, recurrent chest pain requiring additional diagnostics, cardiac rhythm disturbances (tachyarrhythmias, tachycardia, second- or third-degree atrioventricular block), acute heart failure, post-procedural complications such as bleeding or hematomas, severe respiratory disorders, infections or fever, anemia, coagulation disorders (6, 7).

There is a lack of analyses assessing whether an earlier initiation of walking training may further optimize the course of short hospitalizations, improve exercise tolerance more rapidly than traditional programs, and influence patient safety and satisfaction. The present study addresses this gap by proposing a modification of the rehabilitation program starting from day 2 after PCI.

Therefore, the aim of the study was to evaluate the effectiveness of early walking training in patients after non-ST-elevation myocardial infarction (NSTEMI) treated with coronary angioplasty in the first stage of cardiac rehabilitation. It was hypothesized that early walking training in patients with NSTEMI in the first stage of cardiac rehabilitation is safe and brings numerous benefits. It is justified to introduce it on the second day in patients with NSTEMI after coronary angioplasty. The following research questions were asked:

Does early walking training significantly improve exercise tolerance assessed with the 6-minute walk test (6MWT) in patients after myocardial infarction treated with coronary angioplasty?
Can early walking training be an effective method of rehabilitation for patients after myocardial infarction in the first stage of cardiac rehabilitation and become an alternative to standard rehabilitation methods?

### Main hypothesis

Early implementation of walking training starting from the second day after PCI in low-risk NSTEMI patients leads to a significantly greater improvement in exercise tolerance (walking distance, walking speed, METs) without concurrent deterioration of left ventricular function assessed by echocardiography (LVEF, LVEDD, LVESD), compared with a standard rehabilitation program**.**

## Material and methods

The study was carried out as part of a research project titled: “The effectiveness of early walking training in patients after NSTEMI treated with coronary angioplasty in the first stage of cardiac rehabilitation.” The research project received consent from the Bioethics Committee for Scientific Research at the Jerzy Kukuczka University of Physical Education in Katowice – No. 7/2013. Patients were informed about the purpose and the course of the research and gave their informed, written consent to participate in the research. The research period was 12 months; started on 3.01.2014 and finished on 5.01.2015.

### Characteristics of the study groups

Fifty patients after NSTEMI treated with angioplasty with implantation of at least one stent in the Department of Invasive Cardiology, Angiology and Electrocardiology at the Polish-American Heart Clinic were examined. Based on the recruitment and after meeting the inclusion and exclusion criteria, the patients were divided into two groups using a random method (drawing cards with the name of the group – experimental or standard). The study was designed as a single-center, randomized, parallel-group RCT with 1:1 allocation (early walking training starting from day 2 after PCI vs. standard rehabilitation). Patients were consecutively recruited based on predefined inclusion and exclusion criteria and randomly assigned to the study groups using a sealed-envelope randomization method containing the group assignment.

Group 1 (experimental) – 25 patients who underwent 5-day rehabilitation including 6 training units performed twice a day in a 30-meter corridor. On the second day, intensive walking training began.
Control group – 25 patients who underwent standard cardiac rehabilitation (model A). The program also included 6 training units performed once a day in a 30-meter corridor. Fast walking was started on the 4th day.

Characteristics of both groups are presented in Tables 1–3.

The primary endpoint was the change in distance covered in the 6-minute walk test (6MWT) between baseline and the assessment performed after 3 months of intervention. A difference of 50 meters was considered clinically meaningful. The secondary endpoints included changes in heart rate (HR) and left ventricular ejection fraction (LVEF) assessed by echocardiography at 3 or 6 months.

Criteria for including patients in the study:

Non-ST-elevation myocardial infarction (NSTEMI)
Coronary angioplasty (PCI) performed
Complete revascularization
Left ventricular ejection fraction ≥ 45% (to ensure low risk profile)
No left ventricular dysfunction
Age >40 to <70 years
No diseases of the musculoskeletal system

Criteria for excluding patients from studies:

Hemodynamically significant aortic valve stenosis
Valvular defects requiring surgical correction
Ventricular arrhythmias
Acute myocarditis or pericarditis
Uncontrolled hypertension
Acute thrombosis or embolism
COPD in the period of exacerbation
Liver or kidney disease in the period of failure
Complications at the injection site (hematoma, aneurysm, bleeding)

Independent variables:

Type of intervention:
  - Early walking training (starting from day 2 after PCI) – experimental group (Table 5)
  - Standard rehabilitation (training starting from day 4) – control group (Table 4)
Additional independent/control variables (covariates):
  Age, sex, BMI, comorbidities, number of stents, baseline 6MWT parameters (distance, walking speed, METs), baseline echocardiographic parameters (LVEF, LVEDD, LVESD)

Dependent variables:

Exercise tolerance parameters measured during the 6MWT:
  Distance (m), walking speed (m/s), METs, peak heart rate (HR_peak), perceived exertion assessed using the Borg scale
Echocardiographic parameters:
  LVEF (%), LVEDD (mm), LVESD (mm)

Before starting the training, in order to exclude or include the patient in the study based on standard laboratory tests, levels of the enzyme creatine kinase (CK-MB test) and troponins were determined. The decision on safe inclusion in the program was made by the patient's cardiologist based on the results obtained.

## Research methods

The 6-minute walk test (6MWT) was used to assess exercise tolerance before and after the study (8, 9). It was performed in a 30-meter corridor with a flat, even surface, marked every 3 m. Patients read the informed consent form for the examination and signed it. They did not perform any major physical exertion in the 2 h preceding the test. They wore light clothes that did not restrict movement and comfortable shoes with non-slip soles. The 6-minute walk test was performed after a light meal (depending on the time of the test) and taking the patient's medications. Patients walked at their own pace, as fast as they could for 6 min. The test results included the distance the patients walked in a given time in meters, their well-being and a description of their fatigue according to the Borg scale. Recorded parameters: actual distance (m), time (t), average walking speed (km/h), energy expenditure (metabolic equivalent of task – MET). The functioning of the left ventricle was assessed based on an echocardiographic examination performed in the Echocardiography Laboratory of the Department of Invasive Cardiology, Angiology and Electrocardiology of the Polish-American Heart Clinic. The examinations were performed using a Vivid 4-GE Healthcare device equipped with a sector head with a frequency of 2.5 MHz, using one-dimensional M-mode and two-dimensional projections in accordance with the recommendations of the American Society of Echocardiography. After consultation with a cardiologist, three parameters were selected that may determine the effectiveness of the rehabilitation used:

LVEF% – left ventricular ejection fraction (normal >50%),
LVEDD – left ventricular end-diastolic dimension (normal 35–57 mm),
LVESD – left ventricular end-systolic dimension (normal 27–38 mm).

The test was performed on the 5th day of rehabilitation

## Modified walking training scheme

In the first 5 days after the heart attack, rehabilitation consisted of walking at any pace without rest for 12 min (training time was twice as long as the corridor test) or until the training heart rate limit was reached or the patient refused to continue training. The training was carried out under the supervision of a doctor and was preceded by a 5-minute warm-up consisting of dynamic exercises of the upper and lower limbs and coordination exercises.

Each patient in the training group had a training heart rate limit determined by the formula:

heart rate reserve = maximum exercise heart rate - resting heart rate
training heart rate = resting heart rate + 60%–80% heart rate reserve (8).

Walking training took place twice a day in the morning around 10:00 a.m. and in the afternoon around 2:00 p.m.

Day 1–6-minute walk test.

2nd day of walking training:

training heart rate = resting heart rate + 60% heart rate reserve
training heart rate = resting heart rate + 65% heart rate reserve

3rd day of walking training:

training heart rate = resting heart rate + 70% heart rate reserve
training heart rate = resting heart rate + 75% heart rate reserve

Day 4 of walking training:

training heart rate = resting heart rate + 80% heart rate reserve
training heart rate = resting heart rate + 80% heart rate reserve

Day 5–6-minute walk test

Blood pressure was measured before and after the training.

During the examination, the heart rate was monitored with a pulse oximeter. After each training, the distance covered, blood pressure and heart rate, as well as blood oxygen saturation, were recorded.

All patients in both the experimental and control groups completed the study. During the conducted training sessions, no participants reported any discomfort, and no medical intervention was required. Patient satisfaction levels were high.

## Statistical analysis

To perform statistical analyses, computer programs included in the Open Office 4.0.1 package and the StatSoft Statistica 10 package were used.

Descriptive statistics used to characterize the quantitative variables of the study sample:

average measures: arithmetic mean with 95% confidence interval, median, lower and upper quartiles
measures of dispersion: variance, standard deviation with 95% confidence interval, standard error of the mean, range, minimum and maximum values
measure of asymmetry: skewness
measure of dispersion: kurtosis

When verifying statistical hypotheses, the significance level of *ɑ* = 0.05 was adopted. In order to examine the compliance of the empirical distributions of the studied variables with the normal distribution, the Shapiro–Wilk test was used. Before performing the Student's *t*-test for independent variables and analysis of variance, the homogeneity of variances was tested using the Brown-Forsyth test. The statistical tools used to verify statistical hypotheses were the parametric Student's *t*-test (“difference test”) for dependent variables whose distribution is consistent with the normal distribution, the non-parametric Wilcoxon pairwise order test for dependent variables whose distribution is not consistent with the normal distribution, parametric Student's *t*-test for independent variables whose distribution is consistent with the normal distribution, non-parametric Mann–Whitney U test for independent variables whose distribution is not consistent with the normal distribution, non-parametric Spearman's r correlation coefficient, and non-parametric Friedman ANOVA for variables whose distribution is not consistent with the normal distribution or the variances of the studied groups are not homogeneous. Effect sizes were also assessed using Cohen's d, with values interpreted as follows: < 0.2 – small effect; 0.2–0.5 – moderate effect; 0.5–0.8 – large effect; > 0.8 – very large effect. In addition, eta squared (*η*^2^) was calculated as a measure of effect size representing the percentage of explained variance, interpreted as: < 0.01 – small effect; 0.01–0.06 – moderate effect; 0.06–0.14 – large effect; > 0.14 – very large effect.

## Results

### Six-minute walk test

Results of 6MWT in both groups are presented in Table 6.

Results of parameters of 6MWT in both groups are presented in Table 7.

### Echocardiographic tests

Results of echocardiographic test in both groups are presented in Table 8.

Correlation between ejection fraction and 6 MWT is presented in Table 9.

No relationship was demonstrated.

Correlation between left ventricular end-systolic dimension and 6 MWT is presented in Table 10.

No relationship was demonstrated.

Correlation between left ventricular end-diastolic dimension and 6MWT is presented in Table 11.

No relationship was demonstrated.

## Discussion

A meta-analysis conducted by Dibben et al. showed that cardiac rehabilitation programs generally have a limited impact on overall physical activity levels measured in studies—effects were heterogeneous, depending on the measurement method and study quality. The authors emphasize the methodological limitations in this field, including varying activity assessment methods and short observation periods. Even when implemented programs improve exercise capacity (e.g., in the 6MWT), this does not always translate into sustained increases in daily physical activity, which explains the “neutral” results observed in some analyses (10). According to Garcia et al., the walking test has a beneficial effect on cardiorespiratory fitness in older adults. However, the authors highlighted limitations of walking tests as the sole measure of generalized function. In practice, this means that improvement in the walking test does not always correspond to a clinically meaningful improvement in other health domains. Relying exclusively on walking tests to assess the effectiveness of rehabilitation may provide an overly optimistic or misleading picture, making it necessary to use more precise tools and research methods (11).

According to Kristen et al. (12), walking training reduces total mortality and pathology related to cardiovascular diseases. The authors also found a significant correlation between the number of steps taken per day and some risk factors, including: physical fitness, HDL cholesterol level and obesity. To obtain a training effect, it is necessary to achieve a heart rate of ≥70% HRpeak. Grochulska et al. (13) examined 188 patients (133 men and 55 women) participating in an early rehabilitation program after STEMI treated with angioplasty. The applied rehabilitation program resulted in an average increase in 6MWT distance of 52 m (from 483.46 ± 105.15 to 535.53 ± 98.47 m, *p* = 0.0001). This improvement was smaller compared with the present study, in which an increase of 184 m was observed (434.00 ± 88.88 vs. 618.56 ± 79.00 m, *p* < 0.001).With regard to MET values, the authors also reported a statistically significant increase from 6.28 ± 1.98 to 8.50 ± 2.64 (*p* = 0.0001). A similar improvement was observed in the present study (from 8.23 ± 1.48 to 11.31 ± 1.31, *p* < 0.001).

Earlier studies by the same authors (14) likewise demonstrated the effectiveness of the rehabilitation program, confirmed by an increase in 6MWT distance (from 538 ± 80.9 to 593 ± 94.3 m, *p* = 0.0001) and MET values (from 8.4 ± 1.3 to 9.1 ± 2.2, *p* = 0.0001).

The results indicate that in the patients with coronary artery disease, brisk walking is a form of exercise intense enough to induce an adequate training heart rate. Therefore, walking is a form of endurance training often used in cardiac rehabilitation. However, it is worth noting that age-related reduction in leg muscle strength, combined with orthopedic or musculoskeletal limitations, may reduce walking speed in some patients, which in turn may prevent the appropriate training intensity being achieved. Currently, walking training is used in the second stage of cardiac rehabilitation. This study highlights the possibility of using it in patients with a low risk of coronary events in the first stage of cardiac rehabilitation, similarly to 6MWT (15). The 6MWT results show that walking training significantly improved the assessed parameters in the experimental group: distance (*p* < 0.001), speed (*p* = 0.032) and increased energy expenditure (*p* < 0.001). In the control group in which the standard program was used, a significant change was only observed for distance (*p* = 0.044). The intergroup comparison showed significant differences in the assessed parameters in favor of the experimental group (*p* < 0.001). The analysis of hemodynamic indicators assessed during the tests showed a significant change in the increase in peak heart rate values (*p* < 0.001) in the experimental group, which was also associated with an increase in the distance covered and an increase in walking pace. This certainly indicates an increase in the level of exercise tolerance. In the control group, the results improved, but they were not statistically significant. The intergroup analysis showed a significant difference only in HRpeak (*P* < 0.001) with the benefit of the experimental group. The 6MWT is commonly used for indicative assessment of physical performance. Due to its nature, it is much more relatable to daily activity than any of the other exercise tests (16). Available publications show that the 6MWT is a safe test that has been repeatedly performed in patients with cardiovascular problems (17–19). The main advantage is the lack of hardware requirements and its ability to be performed in most health care facilities. A limitation is the restricted possibility of differentiating the causes of reduced exercise tolerance, except for situations where typical chest pain may indicate a cardiac background, and a significant decrease in arterial hemoglobin oxygen saturation may indicate respiratory system dysfunction. With regard to the echocardiographic examination performed before discharge from the hospital, no significant differences were found between the groups in terms of the parameters examined. The analysis of the relationship between selected parameters (LVEF%, LVESD, LVEDD) and the parameters determining the level of exercise tolerance in the 6MWT in both groups also did not show any relationship. Similar results were reported by Wang et al. (20) as well as Soleimannejad et al. (21); however, the latter, in a study conducted in a group of 146 patients, demonstrated a significant increase in LVEF (from 50.54 ± 8.18 to 52.80 ± 8.03, *p* < 0.001).

Similar results were obtained by Wang et al. (20). Such changes were not expected as the assessed echocardiographic parameters were normal and within normal limits. Immediate cardiological intervention when the patient was brought to the clinic allowed us to avoid the risk of impaired contractility of the left ventricle. Moreover, the observation period was relatively short to achieve significant changes in the analyzed parameters. Even if a statistically significant improvement in exercise tolerance occurred within such a short timeframe, achieving meaningful changes in echocardiographic parameters requires a longer duration of rehabilitation. The analysis of the obtained research results indicates the effectiveness of walking training, as soon as the first stage of cardiac rehabilitation, as an alternative to standard rehabilitation methods. Despite the short period of time (5 days), the training showed an increase in exercise tolerance and no negative changes in the hemodynamics of the left ventricle of the heart muscle. Despite the early improvement period in which the training method was used, no negative complications were observed during or after the program. A positive response from patients to the modification was also obtained. Many patients expressed interest in this form of training and expressed their willingness to continue it in the future. Therefore, walking training may be an alternative to standard cardiac rehabilitation within the framework of CCR (Comprehensive Cardiac Rehabilitation), commonly used as part of stage I rehabilitation. Of course, the implementation of this form of physical activity requires long-term analyses in terms of the impact on exercise parameters and, in particular, the morphological and functional elements of the left ventricle. However, with the progress of cardiology, new methods of improvement should be sought (22–24). It may also be useful to analyze the use of walking training in a group of people after a STEMI without left ventricular damage.

## Practical implications

Early walking training, implemented within the first days after NSTEMI in patients post-angioplasty, represents a safe and effective rehabilitation method. It can serve as an alternative to standard Phase I rehabilitation, particularly in centers with limited resources. The simplicity and high patient acceptability of this form of activity promote greater adherence, which may support the long-term maintenance of physical activity and facilitate the integration of walking-based exercises into outpatient and telerehabilitation programs.

## Future research directions

Future studies should assess the long-term effectiveness of early walking training, including its impact on morphological and functional parameters of the left ventricle. It would be valuable to compare this form of activity with other types of training used in early rehabilitation and to analyze its effects in STEMI patient groups. Additional research areas may include the use of telemonitoring tools and the personalization of training loads based on age, muscle strength, and coexisting movement limitations. Studies should also include female patients.

## Study limitations

The study included only 50 patients, divided into two groups of 25, which limits the generalizability of the results and may affect statistical accuracy. The entire training cycle lasted only 5 days, preventing the assessment of long-term effects of walking training, including its influence on left ventricular remodeling, long-term exercise capacity, or disease recurrence. On the other hand, a 5-day hospital rehabilitation period for post-MI patients after successful angioplasty is consistent with ESC standards. All patients were treated in the same interventional cardiology unit, which may further limit the external validity of the findings. The study showed no significant changes in left ventricular parameters between initial and final assessments, likely due to the short observation period; additionally, all analyzed parameters were within normal ranges. Another limitation is that only NSTEMI patients were included, so future studies will also include STEMI patients. It would also be important to analyze outcomes post-discharge, even over a short-term period of 1–3 months.

## Conclusions

Early walking training significantly improved exercise tolerance measured by the 6-minute walk test (6MWT), increasing the walking distance, walking speed, and energy expenditure in the intervention group compared with the control group.
Early walking training implemented during Phase I cardiac rehabilitation demonstrates potential as an adjunctive therapeutic strategy in low-risk patients, although confirmation in larger-scale studies is required.

## Data Availability

The raw data supporting the conclusions of this article will be made available by the authors, without undue reservation.
